# Deep Brain Stimulation in Leigh-Like Syndrome Due to DNM1 Pathogenic Variant

**DOI:** 10.5334/tohm.1017

**Published:** 2025-07-22

**Authors:** Leonel Villa-Villegas, Luz Gabriela Lira-Jaime, Katia Carmina Farías-Moreno, Biagio David González-Ruffino, Alberto Soto-Escageda, Rodrigo Mercado-Pimentel, Carlos Eduardo Piña-Avilés, Carlos Zúñiga-Ramírez

**Affiliations:** 1 Movement disorders and neurodegenerative diseases unit, Hospital Civil de Guadalajara “Fray Antonio Alcalde”, Guadalajara, México; 2 Functional and stereotactical neurosurgery clinic, Hospital Civil de Guadalajara “Fray Antonio Alcalde”, Guadalajara, México; 3 Department of Genetics, Hospital Civil de Guadalajara “Fray Antonio Alcalde”, Guadalajara, México

**Keywords:** Leigh Syndrome, Leigh-like syndrome, Dynamin 1, Mitofusin 2, Hyperkinesias, Deep brain stimulation

## Abstract

**Clinical vignette:**

Leigh syndrome (LS) and Leigh-like syndromes (LLS), now collectively referred to as Leigh Syndrome Spectrum (LSS), encompass a wide range of clinical manifestations, including epilepsy, neurodevelopmental delay, and movement disorders such as ataxia, chorea, and dystonia. Although rare, LSS can present atypical symptoms in certain cases. The primary etiological cause of LSS is genetic, resulting from mitochondrial alterations.

**Clinical dilemma:**

Hyperkinesias in LSS or other mitochondrial disorders can be disabling, leading to a significant reduction in the patient’s quality of life.

**Clinical solution:**

Globus pallidum deep brain stimulation (GPi-DBS) surgery is an effective treatment for hyperkinesias, such as chorea, and dystonia, caused by mitochondrial defects.

**Gap in knowledge:**

Pathogenic DNM1-related mitochondrial disorders with Leigh syndrome phenotype may show long-term improvement of hyperkinetic movements after GPi-DBS.

Leigh syndrome spectrum (LSS) is characterized by significant clinical and genetic heterogeneity. Despite advances in understanding the etiology and pathophysiology of LSS, its geno-phenotypic spectrum is continuously evolving. GPi-DBS may lead to sustained long-term improvement of hyperkinetic movement disorders in patients with LSS caused by pathogenic DNM1 variants. Somehow, drug-resistant epileptic seizures that were part of the clinical spectrum, were also controlled as well.

## Methods

**Figure 1:**
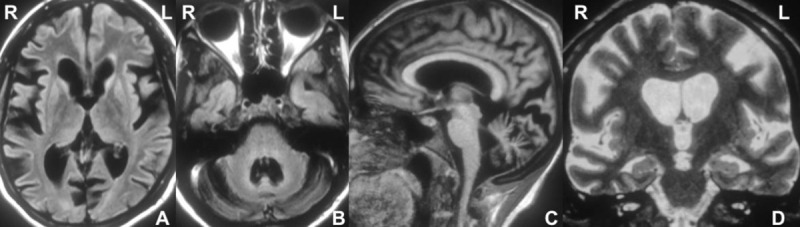
Brain MRI showing generalized atrophy and striatal hyperintensities **(A)**, cerebellar atrophy and a widened fourth ventricle **(B)** and thinning of corpus callosum **(C)**. Hippocampal atrophy and structural changes commonly seen in tauopathies are also seen **(D)**.

**Figure 2:**
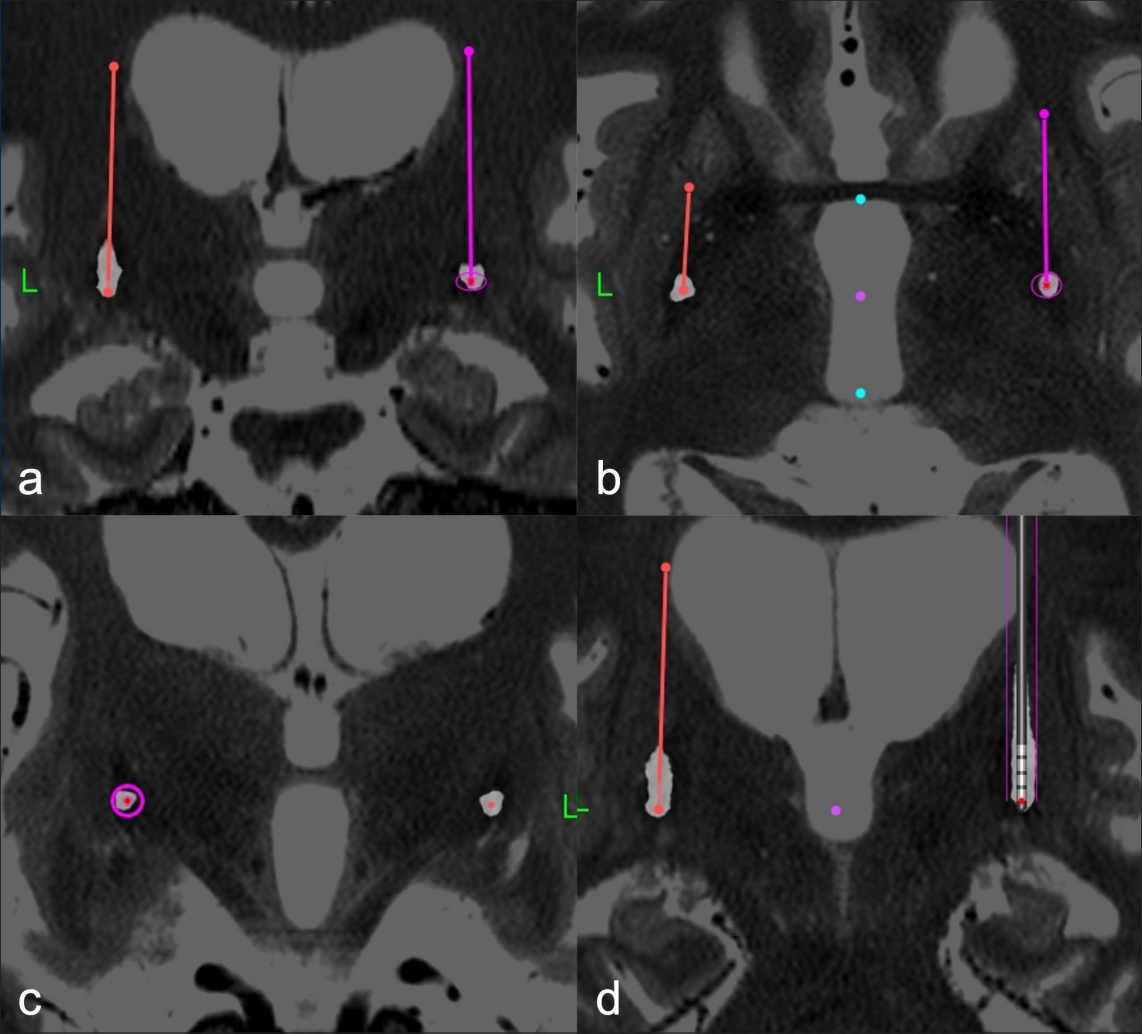
A 3-moths postoperative CT was co-registered with a T2 sequence preoperative MRI (StealthStation 7, Cranial stereotactic software, Medtronic). Coronal **(a)**, axial **(b)**, and probe’s eye **(c)** views of the definitive location of the DBS electrodes, **(d)** shows a parallel reconstruction of the right DBS electrode (Medtronic 3389). The tips of both electrodes were located in the posterolateral region of the ventral GPi, close to the interpallidal lamina. The definitive location coordinates of the electrodes were as follows: rGPi X = 24.7 mm, Y = 1.4 mm, Z = –0.8 mm; lGPi X = 24.5 mm, Y = 1.4 mm, Z = –0.3 mm.

A 33-year-old right-handed woman from a non-consanguineous family presented with drug-resistant epilepsy, generalized dystonia, and neurodevelopmental regression. Generalized tonic-clonic seizures began at age 5 with a frequency of 5–10 episodes per week. The patient showed no perinatal or neurodevelopmental abnormalities until the age of 13, when she began to exhibit gait disturbances due to incoordination, accompanied by lightning-like jerks of the limbs. At the same time, neurodevelopmental regression and the insidious onset of sensorineural hearing loss were noted. At the age of 26, her family observed generalized abnormal postures. Prior to evaluation at our clinic the patient had been managed by pediatric neurology with a diagnosis of drug-resistant epilepsy. An etiological workup was not undertaken, and the patient received multiple antiepileptic drugs, either as monotherapy or in combination, with limited clinical benefit. The patient’s mother had recently died of endometrial cancer; however, she was otherwise not known to have any other medical conditions. The father denied any history of illness at the time of evaluation, and his medical assessment revealed no pathological findings. Initial evaluation revealed multiple non-motor symptoms including apathy, anxiety, inattention, hyperhidrosis, nocturia, and insomnia, all of which began during adolescence. Neurological examination showed a low IQ (see Table A), limited upward gaze, and generalized dystonia involving the trunk and upper limbs, along with an ataxic gait (see Video, part 1). No other neurological deficits were seen. Electroencephalogram displayed generalized spike and wave activity. Nerve conduction studies depicted axonal sensory polyneuropathy. Brain magnetic resonance image (MRI) showed brain and cerebellar atrophy with striatal hyperintensities at FLAIR/T2 sequence (see Figure 1). Whole exome sequencing exhibited a pathogenic heterozygous variant at the Mitochondrial Ribosomal Protein of the Small subunit 34 (MRPS34) gene [NM_001300900.1:c.94C>T p.(Gln32*) exon 1]. Whole genome sequencing depicted a heterozygous pathogenic variant at the Dynamin 1 (DNM1) gene [NM_004408.3:c.824C>T (p.Pro275Leu) exon 6], as well as a heterozygous unknown significance variant at the Mitofusin 2 (MFN2) gene [NM_014874.3:c.1574A>G (p.Asn525Ser) exon 15]. Mitochondrial DNA analysis plus MLPA testing was negative for other variants. Genetic testing on both parents depicted the MRPS34 gene variant in the father only. Further clinical workout did not show further abnormalities, including normal blood lactate levels (1.2 mmol/l, normal values from 0.7 to 2.6 mmol/l). Coenzyme Q10 up to 1200 mg daily showed no improvement. Levetiracetam 1 g TID mildly improved seizures. Bilateral GPi-DBS was performed by the age of 31 (see Figure 2). Both the patient and care providers reported a moderate reduction in dyskinesia amplitude prior to the initiation of stimulation due to lesional effect. Stimulation was initiated in both GPi electrodes two days post-implantation. Bilateral bipolar stimulation (–0 +2) was programmed with an amplitude of 4.5 V, a pulse width of 60 microseconds, and a frequency of 185 Hz. According to the Burke-Fahn-Marsden dystonia motor scale, the patient’s scores were 33.5 preoperatively, 21 at three months, 28.5 at one year, and 12.5 more than two years after bilateral GPi-DBS, improving movement disorders and epilepsy as well (see Table B; Video parts 2 and 3). The subject has been seizure-free since then. It seems that cognitive performance has not worsened after surgery, and dystonia has been under control for more than 2 years after placement of GPi DBS (see Video part 4). Current stimulation parameters are: left side –0 +2, 5 V, 60 microseconds, 185 Hz; right side –0 +2, 4.5 V, 60 microseconds, 185 Hz.

**Table A:** Comparative performance in neuropsychological assessment on April 2019, October 2019 and June 2020. WAIS-IV Wechsler Adult Intelligence Test; IQ Intellectual quotient; HVLT-R Hopkins Verbal Learning Test Revised; ROCF Rey-Osterrieth Complex Figure. *Trail Making Test score reported in seconds. B. Clinical Manifestations and Brain MRI Changes Reported in Pathogenic Variants in DNM1.


A)	*PRE-SURGICAL ASSESSMENT*			*POST-SURGICAL ASSESSMENT 1*			*POST-SURGICAL ASSESSMENT 2*		
		
	RAW SCORE	PERCENTILE SCORE	QUALITATIVE DESCRIPTION	RAW SCORE	PERCENTILE SCORE	QUALITATIVE DESCRIPTION	RAW SCORE	PERCENTILE SCORE	QUALITATIVE DESCRIPTION

***Intelligence (WAIS-IV)***									

**Full scale IQ**	47	<0.1	Deficient – moderate	51	0.1	Deficient – moderate	48	<0.1	Deficient – moderate

**Verbal comprehension**	50	<0.1	Deficient – moderate	56	0.2	Deficient – mild	53	0.1	Deficient – moderate

**Perceptual reasoning**	50	<0.1	Deficient – moderate	56	0.2	Deficient – mild	54	0.1	Deficient – moderate

**Working memory**	52	0.1	Deficient – moderate	52	0.1	Deficient – moderate	49	0.1	Deficient – moderate

**Processing speed**	55	0.1	Deficient – mild	55	0.1	Deficient – mild	50	0.1	Deficient – moderate

***Language***									

**Boston Denomination Test**	17	<5	Deficient	24	<5	Deficient	24	<5	Deficient

**Token Test (verbal comprehension)**	15	–	Moderate impairment	20	–	Moderate impairment	19.5	–	Moderate impairment

**Verbal fluency (animals)**	1	<5	Deficient	2	<5	Deficient	2	<5	Deficient

**Verbal fluency (letter A)**	1	<5	Deficient	2	<5	Deficient	2	<5	Deficient

***Attention***									

Trail Making Test – A*	187	<5	Deficient	300	<5	Deficient	258	<5	Deficient

***Memory***									

**HVLT-R word list total learning**	10	<5	Deficient	14	<5	Deficient	10	<5	Deficient

**HVLT-R word list free recall**	3	<5	Deficient	5	5-10	Borderline	4	<5	Deficient

**ROCF recall**	2	<5	Deficient	2	<5	Deficient	4	<5	Deficient

***Visuospatial skills***									

**ROCF copy**	2.5	<5	Deficient	4	<5	Deficient	7	<5	Deficient

**Clinical Spectrum Reported in Pathogenic Variants in DNM1**									

**B)**	**DNM1**								

**Age of Onset**	Infancy/Early Childhood								

**Clinical Manifestations**	Epileptic Encephalopathy, Neurodevelopmental delay and/or Intellectual Disability, Hypotonia, Spasticity, Myoclonus, Dystonia, Choreoathetosic Movements								

**Brain MRI Abnormalities**	Cerebral volume loss over time, Delayed myelinations, Thin Corpus Callosum [16]								


**Video:** Generalized dystonia with axial involvement, as well as incoordination and ataxia are seen in the patient. Bilateral GPi-DBS was performed, showing improvement in movement disorders since the third month after surgery. Improvement in movement disorders and epilepsy has been maintained for more than 2 years after surgical treatment.

## Discussion

The clinical spectrum now recognized as LSS was first described in 1951 by Denis Leigh [1]. Original description documented the case of a seven-month-old infant presenting with subacute optic atrophy, deafness, global spasticity, extensor plantar responses, rapid progression to coma, and subsequent death. Histopathological examination revealed lesions primarily located in the thalamus, midbrain, pons, and spinal cord, characterized by significant vascular proliferation and gliosis zones in these regions [1].

Currently, LS is known to result from alterations in the mitochondrial respiratory chain, predominantly affecting respiratory complexes I and IV [2], with characteristic histological [1,3] and biochemical changes such as elevated serum lactate levels [2]. Advances in genetic studies have identified multiple causative genes for LS and LLS [2,4,5,6]. Clinically, LSS demonstrates significant heterogeneity in its presentation. However, LS typically manifests with neurodevelopmental delay or intellectual disability, hypotonia and weakness, respiratory dysfunction, epilepsy, and feeding difficulties [7]. Other reported features include movement disorders such as ataxia and dystonia and ophthalmological abnormalities like nystagmus and optic atrophy [8]. The disease can be subclassified based on symptom onset into early-onset syndrome (≤ 2 years) or late-onset (> 2 years) [9], or into typical or atypical syndromes [10], respectively. It is noteworthy that unconventional (atypical) presentations have been characterized by flaccid paralysis, progressive diplegia, central apnea and respiratory arrest, and epilepsy as initial symptoms of the syndrome [10]. Despite advancements in LSS research since its description over 70 years ago, globally accepted diagnostic criteria remain unavailable despite efforts made by Rahman et al. in 1996 [11].

While LS is primarily caused by defects in oxidative phosphorylation, alterations in genes involved in mitochondrial dynamics—such as DNM1—can also result in LS or LLS, now referred as LSS [5]. DNM1 variants have been linked not only to developmental and epileptic encephalopathies [12] or synaptic vesicle cycling disorders [13], but also to the LSS, as reported by several authors [2,5,6,11,14].

DNM1 interacts with other molecules like clathrin and plays a crucial role in neuronal endocytosis. Exclusively located within the central nervous system, it serves an essential function in vesicular fission during endocytosis. Mutations in DNM1 disrupt vesicular trafficking homeostasis and regulation of receptor/protein expression on the plasma membrane [15].

The clinical spectrum associated with pathogenic variants in DNM1 has been previously described [16], encompassing multiple typical manifestations with early onset (< 1 year), such as epilepsy, neurodevelopmental delay/arrest/regression, visual disturbances, movement disorders including choreoathetosis, dystonia and spasticity, and facial dysmorphism. However, atypical phenotypes have also been reported including absence of epilepsy or myoclonic jerks unrelated to epileptic activity.

The characteristic clinical phenotype of DNM1 is largely attributable to mutations in its GTPase domain [16]. DNM1 comprises five domains [15], with different clinical manifestations depending on the affected domain. Brereton et al. [17] reported the first case involving a pathogenic variant within DNM1 PH domain in a patient with neurodevelopmental delay without epilepsy. In turn, although most pathogenic variants are expressed in a heterozygous state, Yigit et al. [12] demonstrated that, on occasion, a biallelic state can lead to the disease. This highlights that clinical manifestations associated with pathogenic variants in DNM1 are highly heterogeneous depending not only on allelic status but also on affected protein domains.

Although no universally accepted diagnostic criteria exist for the LSS, this case supports a LLS associated with a pathogenic DNM1 variant, characterized by mild-neurodevelopmental regression, epilepsy, sensorineural hearing loss, generalized dystonia, ataxic gait, and bilateral basal ganglia hyperintensities on MRI, in the absence of elevated serum lactate levels or response to coenzyme Q treatment [6,11,18]. The c.824C>T variant in the *DNM1* gene results in the p.Pro275Leu amino acid substitution and has been classified as likely pathogenic with moderate evidence according to the VarSome and Franklin databases. It exhibits an extremely low population allele frequency. To date, this variant has not been previously associated with LLS, making this case the first clinical description within the LSS.

The mutation in the GTPase domain of DNM1 alone could account for moderate neurodevelopmental delay (see Table A), epileptic seizures, and dystonia (see Video part 1). Interestingly, while neurodevelopmental delay in these subjects is typically severe, there are reports of atypical cases with mild to moderate neurodevelopmental delay caused by pathogenic variants in the GTPase domain, as observed in our case [19]. It is worth noting that epilepsy has responded satisfactorily to treatment with GPI DBS, despite epilepsy often being resistant to antiepileptic therapies [13,16].

It is crucial to emphasize that certain symptoms cannot be fully attributed to the DNM1 variant, such as axonal sensory polyneuropathy and dysautonomia (manifesting as hyperhidrosis and nocturia in the patient). These symptoms could be possibly associated with the MFN2 variant, as observed in other cases [20,21,22,23]. Nonetheless, the MFN2 c.1574A>C variant is classified as likely benign according to ClinVar [24], based on its frequency in the general population, presence in unaffected individuals, preserved protein function, and lack of association in case-control studies [25,26]. Regarding the heterozygous variant c.94C>T found in the MRPS34 gene, it is well known that compound heterozygous or biallelic states are causal of LS [27]. This variant introduces a premature stop codon, resulting in a truncated protein. In autosomal recessive disorders, mild clinical manifestations have been documented in heterozygous carriers [28]. Therefore, we hypothesize that the identified *MRPS34* variant may act as a disease-modifying gene, contributing to the development of LLS in the patient. However, the way the MRPS34 and MFN2 variants contribute to the patient’s clinical phenotype remains unclear. Despite being a simple heterozygous and likely benign variant respectively, we cannot rule out the possibility that these variants could contribute to the patient’s clinical scenario.

DBS has shown therapeutic benefit in mitochondrial disorders with generalized dystonia, parkinsonism-dystonia, and tremor [29,30,31]. Genotype-phenotype correlations are essential for surgical decision-making, as outcomes vary even within the same gene—for example, ATP1A3-related dystonia may respond differently depending on the clinical presentation [32]. Although reports of GPi-DBS in mitochondrial disorders are limited, emerging evidence—including this case—supports its potential efficacy in patients with pathogenic DNM1 variants.

Finally, we cannot explain the reason for which bilateral GPi-DBS improved not only movement disorders, but also seizures. Previous reports have shown that anterior and centromedian thalamic DBS is effective for epilepsy. However, DBS stimulating the caudate nucleus, cerebellum, hippocampus and subthalamic nuclei could exert benefit as well [33]. We speculate that GPi connections to the thalamus and cortex could be related to this unexpected improvement [34].

To our knowledge, no other LLS due to DNM1 pathogenic variant has been reported involving DBS surgery targeting the internal globus pallidus for dystonia control with a favorable outcome. Surgical approach may be useful for managing hyperkinesias in complex cases such as this one.
